# Sympatry of 2 Hantavirus Strains, Paraguay, 2003–2007

**DOI:** 10.3201/eid1512.090338

**Published:** 2009-12

**Authors:** Yong-Kyu Chu, Douglas Goodin, Robert D. Owen, David Koch, Colleen B. Jonsson

**Affiliations:** Southern Research Institute, Birmingham, Alabama, USA (Y.-K. Chu, C.B. Jonsson); Kansas State University, Manhattan, Kansas, USA (D. Goodin, D. Koch); Texas Tech University, Lubbock, Texas, USA (R.D. Owen); University of Louisville, Louisville, Kentucky, USA (C.B. Jonsson); 1 Current affiliation: Martín Barrios 2230 c/ Pizarro, Barrio Republicano, Asunción, Paraguay.

**Keywords:** Hantaviruses, rodent-borne viruses, viruses, Paraguay, zoonotic pathogens, phylogenetics, Akodon, Oligoryzomys, dispatch

## Abstract

To explore geographic and host-taxonomic patterns of hantaviruses in Paraguay, we established sampling sites in the Mbaracayú Biosphere Reserve. We detected Jaborá virus and Itapúa37/Juquitiba–related virus in locations ≈20 m apart in different years, which suggested sympatry of 2 distinct hantaviruses.

Hantaviruses are rodent-borne viruses that may cause hemorrhagic fever with renal syndrome or hantavirus pulmonary syndrome in humans, although some strains do not cause disease (*1*,*2*). In Paraguay in 1995, Laguna Negra virus carried by *Calomys laucha* (little laucha) caused an outbreak of hantavirus pulmonary syndrome in western Paraguay (*3*).

We have identified 4 additional strains in Paraguay: Alto Paraguay virus harbored by *Holochilus chacarius* (Chacoan marsh rat) in western Paraguay; and Ape Aime virus (AAIV) harbored by *Akodon montensis* (Montane akodont), Itapúa virus strain 37 (IPV37) harbored by *Oligoryzomys nigripes* (black-footed colilargo), and Bermejo virus strain Ñeembucu harbored by *O*. *chacoensis* (Chacoan colilargo) in eastern Paraguay (*4*,*5*). We have continued our surveillance of hantaviruses in the interior Atlantic forests within and near Reserva Natural del Bosque Mbaracayú (RNBM), a World Biosphere Reserve located within Departamento Canindeyú in eastern Paraguay (Figure 1).

**Figure 1:**
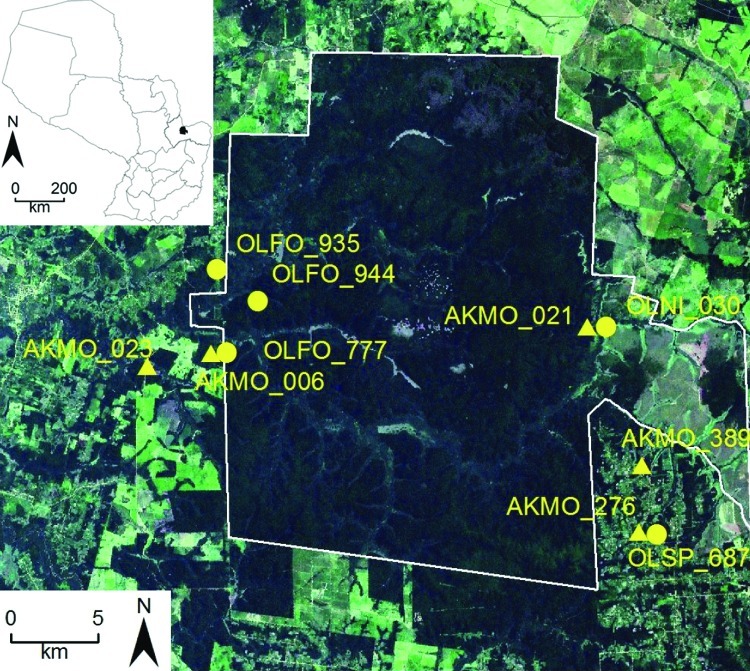
Satellite image of collection sites of hantavirus RNA–positive rodents, including selected Juquitiba virus (circles) and Jaborá virus (triangles) samples, Paraguay, 2003–2007. Inset shows location of showing the study site in Paraguay. OLFO, *Oligoryzomys fornesi*; OLNI, *O. nigripes*; OLSP, *Oligoryzomys* sp.; AKMO, *Akodon montensis*.

## The Study

We established and sampled 10 mark-recapture sites within and adjacent to RNBM during 2003–2007. Sampling of grids depended upon weather conditions, the purpose of the grid, and transitory human settlements. Each mark-recapture grid consisted of an 11 × 11 array of trap stations spaced 10 m apart, each of which had 1 standard live trap (H.B. Sherman Traps, Tallahassee, FL, USA) placed on the ground, and another in branches or vines 2–3 m above ground to capture arboreal species. Grids were sampled for 8 nights, with at least 2 months between sampling sessions. Rodents captured in the mark-recapture grids were individually marked with passive integrated transponder tags, and ≈100 μL of blood was collected from the retroorbital sinus (once per trapping session). Animals were identified to species, age class, reproductive condition, sex, and weight and released.

Rodents were also collected in a series of traplines, each of which contained 50 traps placed ≈10 m apart. Animals collected in traplines were killed, standard collecting information was recorded, and liver, lung, heart, kidney, muscle tissues, and blood specimens were collected. All samples were snap-frozen in liquid nitrogen, transported to the Museum of Texas Tech University (TTU), and stored at –80°C. Standard voucher specimens were prepared from these animals, and have or will be deposited in the Museum of TTU or the Museo Nacional de Historia Natural del Paraguay. All field protocols followed American Society of Mammologists guidelines for the use of wild mammals in research (*6*), and were reviewed and approved by the TTU Animal Care and Use Committee.

A total of 1,150 small mammals from >20 species were captured, including 13 sigmodontine rodent species (1,140 animals) (Technical Appendix). The dominant rodent species in Mbaracayú were *A*. *montensis* (55.7%), *Necromys lasiurus* (hairy-tailed akodont) (10.8%), *C*. *callosus* (big laucha) (6.5%), and *O*. *fornesi* (Fornes’ colilargo) (6.3%).

Antibodies to hantavirus antigens were detected in blood specimens by using an indirect immunofluorescent antibody assay and irradiation-sterilized slides of Vero E6 cells infected with Andes virus as described (*4*). Seven species were antibody positive: *A*. *montensis*, *N*. *lasiurus*, *O*. *fornesi*, *O*. *nigripes*, *Oligoryzomys* sp., *Oryzomys megacephalus* (Azara’s broad-headed *Oryzomys* sp.), and *Oxymycterus delator* (Paraguayan hocicudo). Antibodies to hantavirus antigens were 3× more abundant in blood samples from males than females (Technical Appendix).

Total RNA was extracted from antibody-positive blood clots from mark-recapture samples or lung tissue from killed animals. Nested reverse transcription–PCR was performed to amplify a 371-nt small (S) hantavirus RNA segment (*4*). Hantavirus RNA was detected in 23 *A*. *montensis*, 5 *O*. *fornesi*, 1 *O*. *nigripes*, and 1 *Oligoryzomys* sp. Of these animals, all but 2 *A*. *montensis* were males, which indicated that male rodents play the primary role in maintenance and transmission of hantavirus.

**Figure 2:**
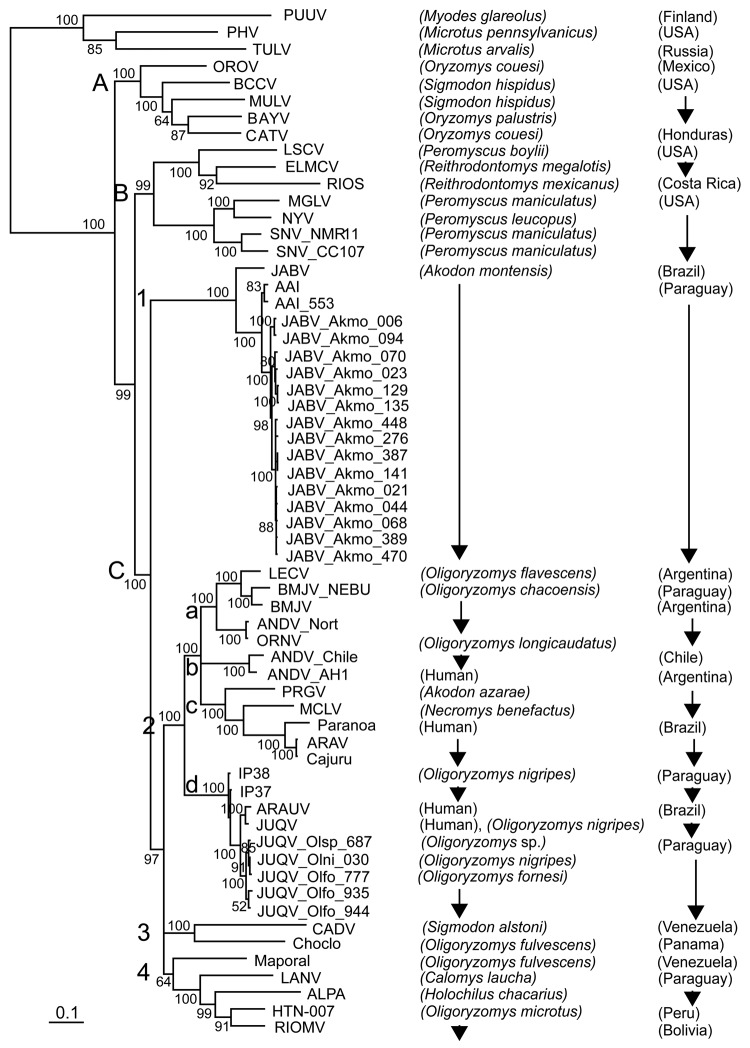
Phylogenetic tree based on Bayesian analysis of the small (S) segment of American hantaviruses, Paraguay, 2003–2007. The tree is based on 1,014 nucleotides of partial S segment of North and South American hantaviruses. Clades (upper case letters), subclades (numbers), and groups (lower case letters) are indicated on the left. Numerical values at the nodes indicate posterior probabilities that supported each interior branch. Scale bar indicates mean number of nucleotide substitutions per site. Sample identifier numbers are the same as in Figure 1. Alignment and editing of nucleotide sequences were conducted by using Vector NTI version 10.3.1 (Invitrogen, Carlsbad, CA, USA). Puumala virus (PUUV) was used as an outgroup for phylogenetic analyses. Estimation of the suitable model of nucleotide substitution and phylogenetic analysis were conducted by using Modeltest version 3.6 (http://darwin.uvigo.es/software/modeltest.html), PAUP* 4.0b10 (http://paup.csit.fsu.edu), and MrBayes version 3.1.2 (http://mrbayes.csit.fsu.edu). A Bayesian analysis was conducted under the general time reversible + invgamma model. Two runs of 4 chains each (1 cold, 3 heated, temperature 0.20) were run for 1 million generations, and trees were sampled every 100 generations. Convergence was assessed by using the average standard deviation in partition frequency values across independent analyses with a threshold value of 0.01; burn-in was set to 25%. Nucleotide sequences of specimens collected at Reserva Natural del Bosque Mbaracayú, Departamento Canindeyú, are indicated by acronyms of related viruses, rodent species, and collection numbers. Hantavirus strain abbreviations and GenBank accession nos. are AAI (Ape Aime) virus from Paraguay [DQ345764]; ALPA (Alto Paraguay) virus from Paraguay [DQ345762]; ANDV_AH1 (Andes virus, strain AH-1) from Argentina [AF324902]; ANDV_Chile (Andes virus, strain Chile 9717869) [AF291702]; ANDV_Nort (Andes virus, strain Nort) from Argentina [AF325966]; ARAV (Araraquara virus) from Brazil [AF307325]; ARAUV (Araucaria virus) from Brazil [AY740633]; BAYV (Bayou virus) from the United StatesA [L36929]; BCCV (Black Creek Canal virus) from the United States [L39949]; BMJV (Bermejo virus) from Argentina [AF482713]; BMJV-ÑEBU (Bermejo virus, strain Ñeembucú) from Paraguay [DQ345763]; Cajuru (Araraquara-like virus, strain Cajuru) from Brazil [EF571895]; CATV (Catacamas virus) from Honduras [DQ256126]; CADV (Cano Delgadito virus) from Venezuela [DQ285566]; Choclo virus from Panama [DQ285046]; ELMCV (El Moro Canyon virus) from the United States [U11427]; HTN-007 virus from Peru [AF133254]; IP37 (Itapúa virus, strain 37) from Paraguay [DQ345765]; IP38 (Itapúa virus, strain 38) from Paraguay [DQ345766]; Jaborá (JAB) virus from Brazil [EF492471]; JUQV (Juquitiba virus) from Brazil [EF492472]; LECV (Lechiguanas virus) from Argentina [AF482714]; MULV (Muleshoe virus) from the United States [U54575]; LANV (Laguna Negra virus) from Paraguay [AF005727]; LSCV (Limestone Canyon virus) from the United States [AF307322]; MCLV (Maciel virus) from Argentina [AF482716]; Maporal virus from Venezuela [AY267347]; MGLV (Monongahela virus) from the United States [U32591]; NYV (New York virus) from the United States [U47135]; OROV (Playa de Oro virus) from the Mexico [EF534077–79]; ORNV (Oran virus) from Argentina [AF482715]; Paranoa virus from Brazil [EF576661]; PHV (Prospect Hill virus) from the United States [U47136]; PRGV (Pergamino virus) from Argentina [AF482717]; PUUV from Finland [X61035]; RIOMV (Rio Mamore virus) from Bolivia [U52136]; RIOS (Rio Segundo) virus from Costa Rica [GenBank U18100 (S segment)]; SNV_CC107 (Sin Nombre virus, strain CC107) from the United States [L33683]; SNV_NMR11 (Sin Nombre virus, strain NMR11) from the United States [L37904]; and TULV (Tula virus) from Russia [Z30941]. OLFO, *Oligoryzomys fornesi*; OLNI, *O. nigripes*; OLSP, *Oligoryzomys* sp.; AKMO, *Akodon montensis*).

A representative sample (15 *A*. *montensis*, 3 *O*. *fornesi*, 1 *O*. *nigripes*, and 1 *Oligoryzomys* sp.) were selected for additional PCR, sequencing, and phylogenetic analysis. PCR-amplified cDNAs of a 1,014-nt amino terminus region of the S segment were purified by agarose gel electrophoresis and cloned into pCRII (Invitrogen, Carlsbad, CA, USA) (*4*,*5*). M13 forward and reverse primers were used for sequencing. For sequence comparison and phylogenetic analysis, sequences of representative New and Old World hantaviruses were obtained from GenBank. Phylogeny reconstruction was conducted by using Modeltest version 3.6 analysis (http://darwin.uvigo.es/software/modeltest.html.), maximum-likelihood estimation, and Bayesian inference (Figure 2).

Bayesian analysis based on the 1,014-nt sequence showed that all sequences from *A*. *montensis* formed a strongly supported clade, which included AAIV-related hantaviruses from Itapúa Department, Jaborá virus (JABV) from southern Brazil, and strains from RNBM in Paraguay (Figure 2, clade C1). Phylogenetic analyses identified 3 subclades representing virus sequences from animals in the RNBM, Itapúa, and southern Brazil. This type of geographic clustering is similar to Sin Nombre–related viruses in deer mice in North America (*7*). All S segment sequences from *A*. *montensis* were closely related, with nucleotide sequence differences between RNBM strains and AAIV and JABV of 4% and 12%, respectively, and derived amino acid differences of 0% or 1%, respectively (Table 1).

**Table 1:** Nucleotide and amino acid sequence similarities of small gene segments among hantaviruses identified in Paraguay and nearby countries, 2003–2007*

Virus	LANV	RIOMV	ALPA	JABV	JABV Akmo_006	AAI	ANDV	BMJV-NEBU	IPV37	JUQV Olfo_777	JUQV	PRGV	ARAV
LANV	–	83	78	75	75	76	79	80	80	80	80	78	78
RIOMV	93	–	81	77	78	77	80	80	79	79	78	79	79
ALPA	92	96	–	77	77	78	78	78	78	77	77	77	78
JABV	85	89	88	–	88	89	76	77	78	77	78	75	77
JABV Akmo_006	86	88	88	99	–	96	76	75	77	76	76	75	76
AAIV	88	90	90	99	99	–	77	76	77	77	77	76	76
ANDV	90	90	89	86	86	88	–	83	82	81	82	82	82
BMJV-NEBU	89	90	88	86	85	88	98	–	84	83	84	82	81
IPV37	90	90	89	86	86	88	96	95	–	95	95	83	81
JUQV_Olfo_777	89	88	87	86	86	87	96	94	100	–	96	82	80
JUQV	90	90	89	86	85	88	96	95	100	100	–	82	81
PRGV	90	90	89	85	84	87	96	95	93	92	93	–	83
ARAV	91	91	90	90	89	90	96	94	94	94	94	96	–

*Values above the diagonal are percentage nucleotide sequence similarities, and values below the diagonal are percentage amino acid sequence similarities. LANV, Laguna Negra virus; RIOMV, Rio Mamore virus; ALPA, Alto Paraguay virus; JABV, Jaborá virus; AAIV, Ape Aime virus; ANDV, Andes virus; BMJV-NEBU, Beremjo virus from Ñeembucú; IPV37, Itapúa virus strain 37; JUQV, Juquitiba virus; PRGV, Pergamino virus; ARAV, Araraquara virus.

In contrast, all virus sequences from *O*. *fornesi*, *O*. *nigripes*, and *Oryzomys* sp. at RNBM formed a strongly supported clade with viruses related to Juquitiba virus (JUQV) from Brazil and Itapúa virus strain 37, which was originally detected in *O*. *nigripes* from Itapúa Department in eastern Paraguay (Figure 2, clade C2d). Nucleotide sequence differences between JUQV strains from RNBM were 0%–2%. Nucleotide sequence differences between JUQV strains from RNBM and Itapúa virus strain 37 or JUQV (Brazil) were 5% or 4%, respectively, and derived amino acid differences were 0% (Table 1). This clade is phylogenetically distinct from viruses that form the *Akodon* *montensis* clade at RNBM and more closely related to Andes (clade C2b) and Bermejo-Ñeembucú (clade C2a) viruses. This finding suggests that spillover infection of JUQV-related viruses is actively occurring among oryzomyine rodent species at RNBM, as reported for other hantaviruses in oryzomyines (*8*) and other rodent hosts of Old World hantaviruses (*9*,*10*). Additional data are needed to determine the primary oryzomyine reservoir of JUQV and to better understand mechanisms by which spillover occurs.

In addition to spillover infection of JUQV among oryzomyine rodents, we identified 2 virus strains (JUQV and JABV) in close proximity (collected ≈20 m apart on the same grid in the same sampling session) on 2 occasions, in sites separated by ≈30 km (Figure 1; Table 2). Thus, these 2 distinct hantaviruses appear to be maintaining a sympatric status across a considerable expanse of landscape, rather than reflecting a temporary or localized phenomenon. We use the term sympatric to underscore that these viruses are in the same community and are near enough (their rodent reservoirs) to interact.

**Table 2:** Incidence of sympatry of 2 hantaviruses and their presumed reservoirs, Paraguay, 2003–2007*

ID no.	Species	Collection date	Collection site	Virus antibody	Virus RNA
JAB_Akmo_006	*Akodon montensis*	2005 Sep 15–18	Mark-recapture	–	–
		2005 Nov 12, 15, 17		+	+
		2006 Feb 27–Mar 6		+	+
JUQV_Olfo_777	*Oligoryzomys fornesi*	2005 Feb 14–16		–	–
		2005 Sep 12		+	+
JAB_Akmo_276	*A*. *montensis*	2007 Jun 12	Trapline	+	+
JUQV_Olsp_687	*Oligoryzomys* sp.	2006 Aug 18		+	+
JAB_Akmo_021	*Akodon montensis*	2003 Sep 12	Trapline	+	+
JUQV_Olni_030	*O*. *nigripes*	2003 Sep 13		+	+

*ID, identification; JAB, Jaborá virus; JUQV, Juquitiba virus.

## Conclusions

Coexistence of hantaviruses in 2 rodent species at mark-recapture sites has been observed (*11*–*13*). Serologic analyses in these studies would not have differentiated whether distinct strains of hantaviruses were co-circulating or active spillover infection was occurring among sympatric rodents at collection sites. Recently, Raboni et al. reported JUQV circulating in 3 sympatric rodent species in southern Brazil and 2 distinct hantaviruses (Jabora and JUQV) in 1 rodent species (*A*. *montensis*) (*14*). We have not detected JUQV in *A*. *montensis* in Paraguay. To address host-jumping of hantaviruses among sympatric rodent species in RNBM and other regions in South America, future longitudinal studies are warranted. Such studies are critical to understanding evolutionary adaptation of hantaviruses in rodents in South America, the ability of these viruses to adapt to new rodent reservoirs, and their emergence and maintenance in the environment.

## Supplementary Material

Technical Appendix Small mammals collected at Mbaracayú Reserve, Paraguay, 2003-2007*
