# Supplementary material for: Sympatry of 2 Hantavirus Strains, Paraguay, 2003–2007
Source: Emerg Infect Dis. 2009 Dec;15(12):1977–80. doi: 10.3201/eid1512.090338 (PMC3044524; doi:10.3201/eid1512.090338)
Supplement: Technical Appendix — Small mammals collected at Mbaracayú Reserve, Paraguay, 2003-2007* [file 09-0338_Techapp-s1.pdf]

Sympatry of 2 Hantavirus Strains, Paraguay, 2003–2007

Technical Appendix

Table. Small mammals collected at Mbaracayú Reserve, Paraguay, 2003–2007*

Species	No. collected			No. positive for antibodies against hantavirus antigens			No. positive for hantavirus RNA		
	M	F	Total (%)	M	F	Total	M	F	Total
Akodon montensis*	319	315	641 (55.7)	50	15	65	21	2	23
Calomys callosus*	41	21	63 (5.5)	0	0	0	0	0	0
Calomys tener	1	1	2 (0.2)	0	0	0	0	0	0
***Calomys* sp.**	5	2	7 (0.6)	0	0	0	0	0	0
Clyomys laticeps	1	0	1 (0.1)	0	0	0	0	0	0
Dasypus novemcinctus	0	0	1 (0.1)	0	0	0	0	0	0
Dasypus septemcinctus	0	1	1 (0.1)	0	0	0	0	0	0
Gracilinanus sp.	1	0	1 (0.1)	0	0	0	0	0	0
Holochilus chacarius	1	0	1 (0.1)	0	0	0	0	0	0
Monodelphis domestica	2	2	4 (0.3)	0	0	0	0	0	0
Mus musculus	0	1	1 (0.1)	0	0	0	0	0	0
Necromys lasiurus	74	50	124 (10.8)	1	2	3	0	0	0
Oligoryzomys fornesi*	41	30	72 (6.3)	4	0	5	4	0	5
Oligoryzomys nigripes*	27	15	44 (3.8)	1	0	1	1	0	1
***Oligoryzomys* sp.**	5	10	15 (1.3)	1	0	1	1	0	1
Oryzomys angouya	14	7	30 (2.6)	0	0	0	0	0	0
Oryzomys buccinatus	0	1	1 (0.1)	0	0	0	0	0	0
Oryzomys megacephalus	33	18	51 (4.4)	1	0	1	0	0	0
***Oryzomys* sp.**	5	13	18 (1.6)	0	0	0	0	0	0
Oxymycterus delator*	25	31	57 (5.0)	0	1	1	0	0	0
Oxymycterus misionalis	3	2	5 (0.4)	0	0	0	0	0	0
Rattus rattus	1	0	1 (0.1)	0	0	0	0	0	0
Scapteromys aquaticus	2	2	4 (0.3)	0	0	0	0	0	0
Unknown	2	3	5 (0.4)	0	0	0	0	0	0
Total	603	525	1,150 (100)	58	18	77	27	2	30

*Species in **boldface** are sigmodontine rodents (family Cricetidae, subfamily Sigmodontinae). Species followed by an asterisk included ≥1 animals of undetermined sex. Thus, the total is greater than males plus females.
